# The Potential Role of Arbuscular Mycorrhizal Fungi in the Restoration of Degraded Lands

**DOI:** 10.3389/fmicb.2016.01095

**Published:** 2016-07-26

**Authors:** Fisseha Asmelash, Tamrat Bekele, Emiru Birhane

**Affiliations:** ^1^Forest and Range Land Biodiversity Conservation Directorate, Ethiopian Biodiversity InstituteAddis Ababa, Ethiopia; ^2^Department of Plant Biology and Biodiversity Management, Addis Ababa UniversityAddis Ababa, Ethiopia; ^3^Department of Land Resources Management and Environmental Protection, Mekelle UniversityMekelle, Ethiopia; ^4^Department of Ecology and Natural Resource Management, Norwegian University of Life SciencesÅs, Norway

**Keywords:** AMF, ecological restoration, facilitation, inoculation, land degradation, mycorrhiza, monoxenic culture, succession

## Abstract

Experiences worldwide reveal that degraded lands restoration projects achieve little success or fail. Hence, understanding the underlying causes and accordingly, devising appropriate restoration mechanisms is crucial. In doing so, the ever-increasing aspiration and global commitments in degraded lands restoration could be realized. Here we explain that arbuscular mycorrhizal fungi (AMF) biotechnology is a potential mechanism to significantly improve the restoration success of degraded lands. There are abundant scientific evidences to demonstrate that AMF significantly improve soil attributes, increase above and belowground biodiversity, significantly improve tree/shrub seedlings survival, growth and establishment on moisture and nutrient stressed soils. AMF have also been shown to drive plant succession and may prevent invasion by alien species. The very few conditions where infective AMF are low in abundance and diversity is when the soil erodes, is disturbed and is devoid of vegetation cover. These are all common features of degraded lands. Meanwhile, degraded lands harbor low levels of infective AMF abundance and diversity. Therefore, the successful restoration of infective AMF can potentially improve the restoration success of degraded lands. Better AMF inoculation effects result when inocula are composed of native fungi instead of exotics, early seral instead of late seral fungi, and are consortia instead of few or single species. Future research efforts should focus on AMF effect on plant community primary productivity and plant competition. Further investigation focusing on forest ecosystems, and carried out at the field condition is highly recommended. Devising cheap and ethically widely accepted inocula production methods and better ways of AMF *in situ* management for effective restoration of degraded lands will also remain to be important research areas.

## Introduction

Ecological restoration has emerged to be the central theme of global environmental policies (Aradottir and Hagen, 2013; Jacobs et al., 2015). Restoration of at least 15% of the world’s degraded ecosystems is one of the 20 2011–2020 targets of the UN Convention on Biological Diversity (CBD, 2010^1^). In 2011, world leaders endorsed the “Bonn challenge” which is a global commitment to restore 150 million hectares of deforested and degraded lands by 2020 (Aradottir and Hagen, 2013). In 2014, the New York Declaration on Forests put forward even a bigger global commitment of restoring 350 million hectares of deforested and degraded lands until 2030 (Jacobs et al., 2015). Most importantly, in 2015, the UN concretized these global commitments by adopting the 2030 Sustainable Development Goals which has one of the 17 targets (Target 15) dealing on ecological restoration (UN, 2015). However, restoration experiences so far show that many restoration projects achieve limited success or fail completely (Thomas et al., 2014) and therefore, extra effort is needed to achieve the huge global restoration commitments put on the table. Here we propose AMF inoculation and *in situ* management for better restoration outcome of degraded lands.

About 93% of flowering plant families (Brundrett, 2009) and 92% of land plant families (Wang and Qiu, 2006) are estimated to have mycorrhizal associations. These associations, based on their structure and physiological relationship with symbionts, are categorized in to seven; of which arbuscular mycorrhiza is one (Brundrett et al., 1996; Barea et al., 2011). Arbuscular mycorrhiza is the most predominant and evolutionarily the ancestor of all the association types (Wang and Qiu, 2006). AMF produce arbuscules, hyphae, and vesicles within host plants’ root cortical cells (Brundrett and Abbott, 2002). However, some species within the family Gigasporaceae do not form vesicles but instead, form auxiliary cells of unknown function (Redecker and Raab, 2006). In few other cases as well, arbuscules develop poorly or may be absent (Koide and Mosse, 2004). AMF are; (1) obligate biotrophs completely depending on host plants for organic carbon (File et al., 2012), (2) evolutionarily intimately associated with plants (Taylor et al., 1995), (3) multiple nucleated, and (4) asexually reproducing eukaryotes (Schüßler et al., 2007).

Arbuscular mycorrhizal fungi are keystone organisms with myriads of ecosystem roles. The external hyphae network (extraradical mycelium) of the fungi permeate in to the microsites of rocks and soils surrounding the plant roots (Finlay, 2008; Barea et al., 2011) increasing the root absorbing surface area 100 or even 1000 fold (Larcher, 1995). Therefore, AMF increase plants’ nutrient and water relation (e.g., Birhane et al., 2012, 2015; Banerjee et al., 2013), and can improve plants’ field survival and establishment (e.g., Pouyu-Rojas and Siqueira, 2000; Habte et al., 2001; Ouahmane et al., 2006; Dag et al., 2009; Kapulnik et al., 2010; Karthikeyan and Krishnakumar, 2012; Manaut et al., 2015). AMF improve soil structure, soil water relation, plants’ tolerance to biotic and abiotic stresses, increase plants’ nutrient supply, plants’ growth, yield and reproductive success and reduce fertilizer requirement (Finlay, 2008; Gianinazzi et al., 2010; Simard and Austin, 2010; Barea et al., 2011; Al-Karaki, 2013; Soka and Ritchie, 2014). AMF influence plant community structure (Van der Heijden et al., 1998; Hartnett and Wilson, 1999; Renker et al., 2004; Heneghan et al., 2008; Lin et al., 2015) and are considered to have a pivotal role in plant community assembly and succession (Janos, 1980; Renker et al., 2004; Kikvidze et al., 2010). Therefore, AMF have significant role in ecological restoration.

The potential role of AMF in ecological restoration has been well recognized even before restoration ecology emerged as a scientific field of study (see Janos, 1980 and the references there). However, as of yet, there is no report available to confirm that AMF inoculation has grown to be a biotechnological tool that is widely applicable in ecological restoration. Review articles dealing on the subject are also very few. To our knowledge, those review articles that dealt on the subject are Skujins and Allen (1986), Brundrett and Abbott (2002), Jeffries et al. (2002) and Renker et al. (2004). Other reviews (e.g., Perry et al., 1987; Quoreshi, 2008; Sanon et al., 2010; Al-Karaki, 2013) did not deal on AMF specifically and the one by Koide and Mosse (2004) allocated some paragraphs for the topic. Therefore, although there is a large number of articles and sufficient knowledge on the ecosystem role of AMF, their role in the restoration of degraded lands is relatively little reviewed. Hence, the purpose of this review article is to gather data from published articles and assess the effects AMF have on measurable ecological restoration attributes and ecological processes.

## Features of Degraded Lands

There is no single internationally approved definition of land degradation (World Resource Institute, 2015^2^). However, land degradation is often defined as a long-term loss of ecosystem function and productivity caused by disturbances from which the land cannot recover unaided (e.g., Bai et al., 2008). Meanwhile, reduction in net primary productivity has commonly been used to measure the level of land degradation and restoration (Bai et al., 2008). The Society for Ecological Restoration (SER), however, recommends nine attributes to measure restoration success (SER, 2004). Earlier, Aronson et al. (1993) adapted Odum (1969) succession traits to formulate their own restoration attributes. The SER restoration attributes are excellent parameters (Ruiz-Jaen and Aide, 2005), however, it is the Aronson et al. (1993) restoration attributes that have commonly been used by restoration ecologists (Choi, 2004; Ruiz-Jaen and Aide, 2005). Therefore, in this article, the attributes listed by Aronson et al. (1993) are adapted and used to safely characterize degraded lands (**Table 1**).

**Table 1:** Features of degraded lands compared to reference climax ecosystems (Based on Aronson et al., 1993).

Structural indicators of degraded lands	Functional indicators of degraded lands	
Low total plant cover	Low biomass productivity	

Low perennial and annual plant species richness	Low soil organic matter	

Low aboveground phytomass	Poor soil water relation	Lowered soil water reserves
		
Low beta diversity (species turnover along environmental gradient)		Low coefficient of rainfall efficiency (the amount of water infiltrating to middle and deep soil layers)
		
Decreased life form spectrum (Decreased number of species with different modes of adaptation)		Reduced length of water availability period

Reduced number of keystone species	Low rain use efficiency (RUE)	

Low soil microbial biomass	Poor nutrient cycling index (the ratio of the amount of nutrients mainly N&P recycled to the amount leaching or lost)	

Low soil microbial diversity	Low nitrogen use efficiency (NUE)	


Degraded lands are characterized by low levels of AMF abundance and diversity. An experiment carried out in Brazil (Cardozo-Junior et al., 2012) compared the abundance and diversity of AMF on lands of differing degradation levels and also determined the same on a young restoring site. The result of the observation clearly showed that as the scale of degradation increases, the abundance and diversity of AMF reduces and when restoration presumes both AMF abundance and diversity increase (Cardozo-Junior et al., 2012). Elsewhere also, it was reported that an effective exclosure increased AMF abundance (Birhane et al., 2010). These are in agreement to the remarks by Abbott and Robson (1991) and Schnoor et al. (2011) which indicated that although AMF are ubiquitous, the very few conditions where a natural ecosystem can be devoid of AMF is in areas that are severely eroded or disturbed.

A greenhouse experiment with simulated erosion was able to demonstrate that erosion of soil beyond 7.5 cm could make soil loose AMF completely (Habte, 1989). Likewise, Jasper et al. (1989) were able to experimentally determine that, while AMF maintained their infective potential in extremely dry soil conditions, their infective potential was significantly lowered when the soil was disturbed. Hyphae are important source of inoculum but are highly susceptible to disturbance and hence, disturbance leads to lowered infective potential of AMF (Brundrett and Abbott, 2002).

Furthermore, several studies conducted in agricultural fields have shown that disturbance not only reduces AMF abundance, diversity and infectivity but also results in drastic shift in the AMF community (Schnoor et al., 2011). Most species of the most common AMF families (Glomeraceae, Acaulosporaceae, and Gigasporaceae) have distinctive biomass allocation strategies whereby species of the Glomeraceae allocate most of their biomass in the intraradical hyphae while species of the Gigasporaceae allocate most of their biomass in the extraradical hyphae and species of the Acaulosporaceae produce low biomass both intra and extraradically (Maherali and Klironomos, 2007). Similarly, these distinctive fungal groups have distinctive life history with most species of the Glomeraceae being ruderals while that of Gigasporaceae and Acaulosporaceae are competitors and stress tolerators respectively (Chagnon et al., 2013). Ruderal AMF species are disturbance tolerant since they have shorter extraradiacal mycelium and have the following life history strategy viz., grow faster, have short life cycle and invest earlier and more abundantly in spore formation, fuse fragmented hyphae more readily, and form cross-walls that enable infected root pieces and severed hyphal fragments to heal and re-colonize host roots (Chagnon et al., 2013). Meanwhile, AMF communities of disturbed sites are characteristically dominated by disturbance tolerant species of the family Glomeraceae and more specifically the genus Glomus (Chagnon et al., 2013).

The number of surviving propagules of AMF in soils also declines with time in the absence of host plants (Brundrett and Abbott, 2002). Alexander et al. (1992) have reported that heavy logging in a Malaysian forest significantly reduced (75% reductions) the abundance and infectivity of AMF propagules. Therefore, considering the fact that land degradation significantly reduces plant cover, increases soil disturbance and erosion, low levels of AMF abundance, diversity and infective potential can be considered as a peculiar feature of degraded lands. Degraded lands are also prone to invasion by exotic alien species. This is because, the low level of native plants diversity can potentially provide vacant niche for invasives (Mack et al., 2000).

## What is Ecological Restoration?

Ecological restoration, according to Hobbs et al. (2007) is a process of assembly and succession mediated by disturbance. Succession refers to the more or less regular and predictable replacement of seral communities while community assembly refers to the species dynamics of each seral community. Community assembly is typically viewed as a hierarchical process with local species assemblages representing subsets of a larger species pool (Kikvidze et al., 2015). There are three level filters that result in a particular seral community assemblages viz. (1) speciation, extinction and migration, (2) dispersal, and (3) habitat filters (abiotic factors) and biotic filters like competition and facilitation (Gotzenberger et al., 2011). A typical community assembly was observed by Gleason (1927) whereby ponds at similar locality with similar environmental condition formed different kinds of wetland communities.

Restoration strategies of degraded terrestrial systems usually center on manipulation of species order of arrival and modification of filters to accelerate succession and/or jump start succession (Young et al., 2001; Hobbs et al., 2007; Gómez-Aparicio, 2009). The seral community assemblage is very important in ecological succession since it can determine the latter seral community assemblage and hence, succession trajectory. Egler (1954), argued that ecosystem development can be accelerated by controlling initial species composition and succession to achieve the desired end point. Some field observations (e.g., Cortines and Valcarcel, 2009) have shown that this has practical application in ecological restoration. This phenomenon is known as the priority effect (Young et al., 2005; Zedler, 2005). Hence, designing initial species composition and ensuring their survival and establishment is an important step in ecological restoration (Angelini et al., 2011). With the growing appreciation to plant–plant facilitation interaction and due to better adaptation to resource limited conditions, early/mid successional shrubs are gaining preference to late successional tree/shrubs to startup restoration process of degraded lands (Gómez-Aparicio, 2009; Padilla et al., 2009).

Restoration ecologists not only provide appropriate conditions for desired species to establish but they also, in the meantime, devise ways of preventing the establishment by invasives (Zedler, 2005). Disturbance is also an important factor in ecological restoration since it can modify filters and community assemblage (Choi, 2004). Restoration ecologists have observed that some low level of natural disturbance (e.g., logging, fire, flooding, etc.) can enhance biological diversity and hence, ecological restoration (Palmer et al., 1997).

Based on the scope and complexity of intervention, ecological restoration ranges from species reintroduction to population restoration to community restoration (Young et al., 2001). Based on the restoration goal, it ranges from reclamation to rehabilitation to true restoration. An ecosystem that is slightly disturbed can restore back to its pre-disturbance status (true restoration). As the magnitude of disturbance increases the return to pre-disturbance status may be impossible and hence, return to an intermediate successional status of the given community (an alternative steady state) may be achieved (rehabilitation). When the disturbance is severe, the threshold of irreversibility is passed and the return to pre-disturbance community status or intermediate successional status will be completely impossible and hence, restoration can only result in a novel community stature (reclamation) (Aronson et al., 1993). In the advent of climate change, to have reclamation as a restoration goal is considered to be relevant since novel climatic conditions are anticipated in the future (Choi, 2004).

Considering the wide range of concepts embedded in ecological restoration, as shown above, a comprehensive definition is crucial. Hence, the SER defined ecological restoration as, the process of assisting the recovery of an ecosystem that has been degraded, damaged, or destroyed (SER, 2004). This is the most widely accepted definition of ecological restoration (Harris et al., 2006; Higgs et al., 2014). Likewise, for this article, this definition is adopted.

In tropical lands ecological restoration, tree planting (Lamb et al., 2005; Holl et al., 2010; Aerts and Honnay, 2011) and re-vegetation/reforestation (Cortines and Valcarcel, 2009; Al-Karaki, 2013) are known to be the most effective and widely used biological measures. Accordingly, in this article ecological restoration is considered to be the re-vegetation of degraded sites mainly through tree/shrub planting.

## AMF and the Mycorrhizosphere Ecology

The rhizosphere is a narrow zone of soil affected by the presence of plant roots (Hrynkiewicz and Baum, 2011). It is extremely important and active area for root activity and metabolism (Saharan and Nehra, 2011). Roots release a multitude of organic compounds (e.g., exudates and mucilage) derived from photosynthesis and other plant processes making the rhizosphere a hot spot of microbial activities mainly that of fungi and bacteria (Hrynkiewicz and Baum, 2011). The physical, chemical and biological environment of the rhizosphere is hence, clearly distinct from the bulk soil (Barea et al., 2002).

Similarly, the rhizosphere of the mycorrhizal plant can be referred to as the mycorrhizosphere (Barea et al., 2002). Mycorrhizosphere comprises both the root and hyphae influence zones or the rhizosphere and hyphosphere (Timonen and Marschner, 2006). Mycorrhizal hyphal growth in soils is extensive, with mycelial lengths reaching 111 m cm^-3^ or 0.5 mg g^-1^ or 900 kg ha^-1^ of soil (Simard and Austin, 2010). Hence, the mycorrhizosphere provide a critical link between plants, other microorganisms and the soil (Hrynkiewicz and Baum, 2011).

Intricate interactions take place within the mycorrhizosphere. The most important ones could be interactions between; AMF and the plant, AMF and bacteria, AMF and other fungi, and among AMF themselves. These interactions commence when plant roots exude strigolactones (SLs) (Parniske, 2008; Gutjahr, 2014). Under phosphate or nitrogen limiting conditions plants exude elevated amounts of SLs into the rhizosphere (Gutjahr, 2014). SLs are carotenoid-derived plant hormones (Gutjahr, 2014) that induce AMF spore germination and hyphal branching (Parniske, 2008). They are also known to induce seed germination in parasitic plants, such as *Striga* (Parniske, 2008) and are also involved in suppression of shoot branching and shaping root architecture (Gutjahr, 2014).

The AMF on their part, produce mycorrhiza (Myc) factors. Myc-factors induce calcium oscillations in root epidermal cells and also activate plant symbiosis-related genes (Parniske, 2008). Then the AMF form special type of appressoria called hyphopodia which develops from mature hyphae (Parniske, 2008). As a consequence of sequential chemical and mechanical stimulation, plant epidermal cells produce a pre-penetration apparatus (PPA) (Parniske, 2008). Subsequently, a fungal hypha that extends from the hyphopodium enters the PPA, which guides the fungus through root cells toward the cortex. The fungus then leaves the plant cell and enters the apoplast, where it branches and grows laterally along the root axis (Parniske, 2008; Gutjahr, 2014). These hyphae induce the development of PPA-like structures in inner cortical cells, subsequently enter these cells, and branch to form arbuscules (Parniske, 2008). Upon getting nourished via the arbuscules the fungi will develop extraradical mycelium whose leading tips form new spores to continue the lifecycle of the fungi (Parniske, 2008). Vesicles, which are proposed to function as storage organs of the fungus, when applicable, are formed in the apoplast (Parniske, 2008).

Arbuscular mycorrhizal fungi have a significant role in plants’ P nutrition and sometimes, 100% of the P may be provided by the AMF (Smith et al., 2011). In return, plants allocate, according to most authors, 4–20% of the photosynthate to the AMF (Lerat et al., 2003). It has been shown that plants preferentially allocate more carbon in favor of the more beneficial fungi (e.g., Bever et al., 2009). Moreover, plants allow the arbuscules to live in their cells as long as the AMF is delivering phosphorous and maybe other nutrients efficiently (Parniske, 2008). The observation that mutation of the arbuscule specific phosphate transporter PT4 results in premature degradation of arbuscules suggests that the lifetime of arbuscules is influenced by their ability to deliver phosphate and probably other nutrients (Parniske, 2008). This provides the plant with a means to maintain efficient arbuscules and penalize inefficient ones with early degradation. Conceptually, this mechanism allows the plant not only to discriminate between efficient and inefficient fungal species but also allows to remove potentially ‘good’ fungal symbionts that are attached to a poor phosphate source. This concept allows fungal clones and species to compete for arbuscule formation, which allows succession in an established root system (Parniske, 2008). Meanwhile, the formation of fungal colonization structures and the extent of root colonization are largely under plant control (Gutjahr, 2014).

Arbuscular mycorrhizal fungi are known to play role in plant nutrition as long as they collaborate with other soil microbes. It was experimentally proven that mechanisms underlying the increased P-uptake in arbuscular mycorrhizal plants were solely due to AMF synergistic interactions with P-solubilizing microorganisms and/or greater soil volume explored by the AMF hyphae (Antunes et al., 2007). Otherwise, the AMF unlike ectomycorrhiza are not able to neither solubilize phosphate nor decompose organic matter (Simard and Austin, 2010). The well-known activities of nitrogen-fixing bacteria and P-solubilizing microorganisms improving the bioavailability of the major plant nutrients N and P are very much enhanced in the mycorrhizosphere where synergistic interactions of such microorganisms with mycorrhizal fungi have been demonstrated (Barea et al., 2002). In particular, mycorrhizal inoculation improved the establishment of both inoculated and indigenous P-solubilizing rhizobacteria and, again P-solubilizing rhizobacteria usually behave as mycorrhiza-helper-bacteria, promoting mycorrhiza establishment by both the indigenous and the inoculated mycorrhizal fungi (Barea et al., 2002).

Arbuscular mycorrhizal fungi also interact with decomposer fungi (Soka and Ritchie, 2014) and phosphate solubilizing fungi (PSF) synergistically (Osoria and Habte, 2001). Accordingly, presence of mycorrhizal fungi is known to alter the rates of above and below ground litter decomposition due to chemical changes in the roots and interactions with the decomposer fungi (Soka and Ritchie, 2014). PSF were also observed to have lesser effect in plant nutrition when applied alone and maximum effect took place when both AMF and PSF were inoculated showing the synergistic interaction between the AMF and PSF (Osoria and Habte, 2001). In the meantime, AMF are also known to have antagonistic relationship with root pathogens (Soka and Ritchie, 2014) and even leaf pathogens (Parniske, 2008).

Arbuscular mycorrhizal fungi may also interact with each other synergistically. It was experimentally found out that AMF effects are greater when AMF consortia inoculums are applied than single AMF (Banerjee et al., 2013). After long years of observation, Barea et al. (2011) concluded that the use of native AMF consortia has the maximum effect. A meta-analysis on 306 studies also indicated that plant response was substantially lower when plants were inoculated with single AMF species, compared with inoculations with multiple AMF species (Hoeksema et al., 2010). This could be due to synergistic interaction between the various AMF species. Different species of AMF have different hyphal growth patterns, anastomoses and branching frequencies (Parniske, 2008). These differences probably reflect different strategies and the occupation of different niches within the soil (Parniske, 2008).

## AMF and Measurable Restoration Attributes

Improved plant fitness (survival, growth and reproduction), nutrient uptake and accumulation, tolerance of adverse conditions (biotic and abiotic stresses) and altering plant community structure [competition/facilitation, diversity (richness and evenness) and succession] and that of animal communities (Direct effects on organisms which feed on fungi and indirect effects due to changes in plant fitness) were identified to be the pivotal role AMF play in ecological restoration (Brundrett and Abbott, 2002).

Based on Aronson et al. (1993), the functional and structural attributes to measure ecological restoration include; soil organic matter, soil water relation, nutrient cycling index, plant diversity and soil microbial diversity and abundance, and plant productivity. Therefore, the role of AMF in soil organic matter content, soil water relation, nutrient cycling index, plant stress tolerance, plants survival, establishment and growth on degraded soils, plant diversity, soil microbial diversity and abundance, and plant succession, competition/facilitation and productivity is highlighted below.

### AMF Improve Soil Aggregation; Hence Increase Soil Organic Matter and Soil Water Relation

Fungi and most importantly AMF may be the most effective soil organisms in stabilizing soil structure (Augé, 2004). AMF hyphae grow into the soil matrix to create the skeletal structure that holds primary soil particles together to form soil aggregates (Augé, 2004; Al-Karaki, 2013). AMF also improve soil aggregation by influencing bacterial communities that can improve soil aggregate formation (Rilling, 2004). Furthermore, the dead AMF hyphae produce glomalin which is hydrophobic stable aggregate former (Barea et al., 2002; Simard and Austin, 2010). Hence, AMF increase both soil aggregation and stability. AMF may stabilize soils up to 5 months after their host’s death (Soka and Ritchie, 2014).

Meanwhile, as a result of the significant amount of mycorrhiza derived soil carbon (Rilling, 2004) and improved soil aggregation and stability, AMF increase soil organic matter content and stability (Rilling, 2004; Leifheit et al., 2014). Improved soil aggregation also increases soil water relation. It was observed that a naturally non-mycorrhizal plant planted in mycorrhizal soils tolerated drought more than the ones planted in a non-mycorrhizal soils indicating that AMF hyphae improves water holding capacity of soils (Marschner, 1995).

### AMF Improve Plant Nutrition and Nutrients Cycling Index

The most important role of AMF is their role in phosphorous nutrition (Skujins and Allen, 1986). There are also data indicating that AMF can transfer nitrogen from one plant to another (e.g., Requena et al., 2001), increase the utilization of different forms of nitrogen by plants and can also take up nitrogen directly and transfer it to host roots (Govindarajulu et al., 2005). However, there is considerable doubt as to the cost-benefit of AMF in plant N nutrition (Smith and Smith, 2011). Although few data exist, AMF were observed to improve potassium nutrition in plants (Dag et al., 2009; Garcia and Zimmermann, 2014). AMF can also increase the uptake of other macro and micro nutrients by plants (Birhane et al., 2012). Generally, the external mycelium of AMF establishes an underground network that links the different plants and hence sequester carbon, nitrogen, and phosphorous and also allow the transfer of these nutrients among plants (Rodriguez-Echeverria et al., 2007). These important roles of AMF therefore play great role in nutrient cycling where the need for further nutrient inputs is significantly reduced (Gianinazzi et al., 2010; Al-Karaki, 2013).

Arbuscular mycorrhizal fungi not only improve nutrient cycling but also reduce nutrient leaching from the soil (Rodriguez-Echeverria et al., 2007). In a comprehensive assessment done by Bender et al. (2015), it was possible to determine the role AMF have in nutrient cycling and leaching. Accordingly, it was determined that while AMF inoculation increased nutrient uptake by plants it also reduced leaching of dissolved organic N and un-reactive P (Bender et al., 2015).

### AMF Increase Plants’ Abiotic Stress Tolerance

It was, several times, demonstrated that AMF can increase plants’ tolerance to drought and salinity (Al-Karaki, 2013). AMF are also known to alleviate heavy metal stress in plants (Leyval et al., 1997; Hildebrandt et al., 2007; Soares and Siqueira, 2008; Amir et al., 2013). By inoculating plants with drought tolerant AMF, up to 42% reduction in plants’ water requirement could be achieved (Gianinazzi et al., 2010). Also, Navarro et al. (2013) found out that, *Citrus* rootstocks inoculated with AMF showed significantly increased growth than non-inoculated individuals despite the fact that inoculated individuals were irrigated with saline water and the non-inoculated ones got irrigated with non-saline water.

The mechanism by which AMF increase plants’ tolerance to drought, salinity and heavy metal stresses is mainly nutritional (Marschner, 1995; Soares and Siqueira, 2008; Birhane et al., 2012; Al-Karaki, 2013; Navarro et al., 2013). Soares and Siqueira (2008) demonstrated that both P fertilization and AMF inoculation of plants significantly improved plants’ growth on heavy metal polluted soils. Hence, they concluded, AMF increase plants’ heavy metal stress tolerance mainly through P nutrition.

The non-nutritional mechanisms by which AMF increase plants’ tolerance to drought include; hormonal changes, hyphal soil improvement (delayed soil drying), hyphal ability to scavenge water from micro-pores, increased plants’ photosynthetic rate, and accumulation of compatible osmolites (Marschner, 1995; Birhane et al., 2012; Al-Karaki, 2013). Likewise, immobilizing heavy metals in their biomass mainly cell wall, vesicles and in the glomaline is the non-nutritional mechanism by which AMF improve plants’ tolerance to heavy metals stress (Hildebrandt et al., 2007).

The positive AMF effects on plants’ drought tolerance can improve plants’ salinity tolerance as well. Better water intake by plants can effectively dilute salts within the plants’ cells (Larcher, 1995). Other non-nutritional mechanisms by which AMF improve plants’ salinity tolerance include; exclusion of salt from plant cells by accumulating the salt within the fungal hyphae, production of enzymes involved in antioxidant defense, and change in cell wall elasticity and membrane stability (Al-Karaki, 2013).

### AMF Increases Plants’ Resistance and Tolerance to Pathogens and Herbivores

There are several published articles showing the role of AMF in increasing plant tolerance against biotic stressors. The meta-analysis of 144 published papers clearly reveals that (Yang et al., 2014). Considering the role AMF have in bioprotection, Gianinazzi et al. (2010) described AMF as ‘health insurance’ of plants. One mechanism by which AMF increase plants’ pathogen tolerance could be the synergistic interaction of AMF have with plant growth promoting rhizobacteria (PGPR). PGPR have a very well documented role in plant pathogen inhibition (Figueiredo et al., 2010). The fact that AMF stimulate the synthesis of plant secondary metabolites (Gianinazzi et al., 2010) may also explain why AMF inhibit herbivory. Plants’ secondary metabolites are known to have role in plants’ defense against herbivores (Larcher, 1995). The other reason by which AMF increase plants’ herbivory tolerance is compensatory growth. A microcosm investigation revealed that mycorrhizal plants did not show a reduction in total above ground biomass despite their leaves being fed by grasshoppers indicating that mycorrhiza helped the plant to compensate in growth after herbivory (Kula et al., 2005).

### AMF Increase Tree/Shrub Seedlings Growth, Productivity, Field Survival and Establishment on Degraded Lands

**FIGURE 1:**
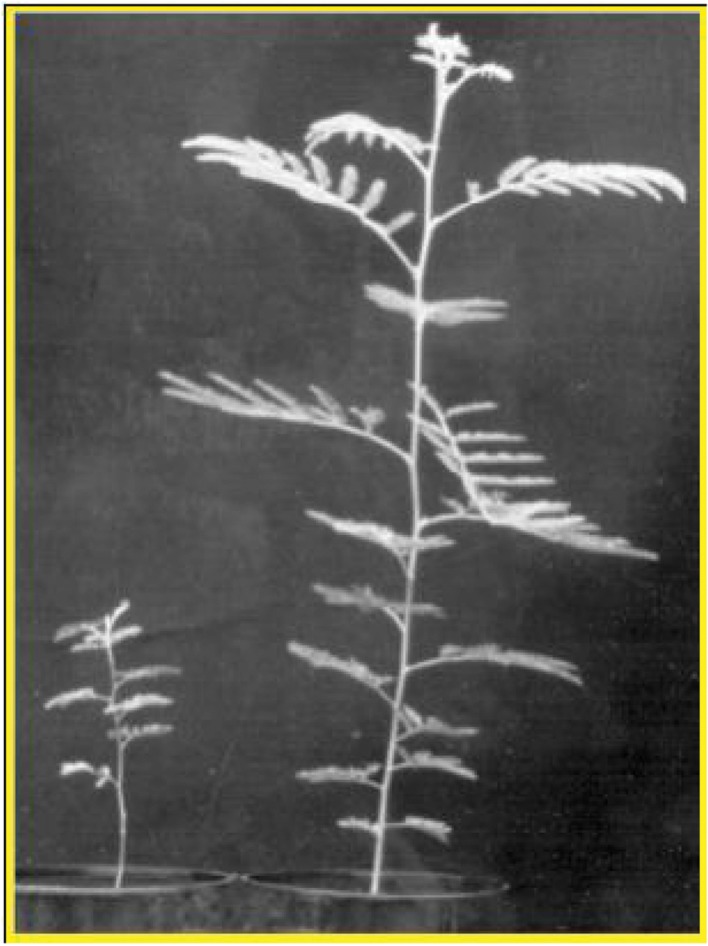
***Acacia koa* A. Gray grew significantly tall in a low-P soil when inoculated with AM fungus (adopted from Miyasaka et al., 2003)**.

Lekberg and Koide (2005), carried out a meta-analysis based on 290 published experiments to determine the role of AMF on plant growth and productivity. The analysis also determined the effects of three common AMF management methods; inoculation, short fallow, and reduced soil disturbance. The result of the meta-analysis revealed that AMF generally increase individual plant’s growth and productivity. Inoculation and short fallow resulted in significantly positive effects on plants’ growth and productivity (Lekberg and Koide, 2005). A recent meta-analysis on 304 papers also concluded that AMF inoculation increases the growth and productivity of plants grown alone (Lin et al., 2015). A similar result was also reported by Birhane et al. (2014). Huante et al. (2012) also did experiment on six tree species and found out that AMF inoculation has significant effect on seedlings growth and most significantly slow growing tree species. **Figure 1** below shows how AMF inoculation can significantly increase tree seedlings growth.

Tree survival and field establishment is an important factor in the restoration of degraded lands. Hence, AMF are important since they can significantly improve tree seedlings field survival and establishment. Pouyu-Rojas and Siqueira (2000), Habte et al. (2001), Ouahmane et al. (2006), Dag et al. (2009), Kapulnik et al. (2010), Karthikeyan and Krishnakumar (2012), and Manaut et al. (2015) have demonstrated the positive effect AMF have in these regards. Pouyu-Rojas and Siqueira (2000) investigated the AMF effect on seven tree species seedlings survival and establishment on degraded pot soils. They found out that AMF inoculation in the nursery or during transplanting have equally significantly positive effect on trees survival and establishment. Later on Habte et al. (2001) determined the effect AMF nursery inoculation has on field establishment of *Acacia koa* and accordingly, AMF was shown to improve transplanted tree seedlings growth and establishment by increasing seedlings P nutrition. The role of native AMF inoculation was also demonstrated to have a significant positive effect on the field survival and establishment of *Cupressus atlantica* Gaussen seedlings on a degraded Moroccan field site (Ouahmane et al., 2006).

Similarly, Kapulnik et al. (2010) determined AMF nursery inoculation effect on seedlings field establishment and growth of *Olea europaea* L. Meanwhile, they were able to observe that AMF inoculation improved seedlings field performance significantly and most importantly for the first 2.5 years from transplanting. They also observed that AMF effect decreased with increasing seedlings age. Karthikeyan and Krishnakumar (2012) also determined AMF effect on survival and establishment of *Eucalyptus tereticornis* Sm. on pot soil of highly degraded origin (mine spoils). Meanwhile, they were able to observe that AMF inoculation almost doubled seedling survival and significantly increased establishment. Recently, Manaut et al. (2015) demonstrated that native AMF consortia inoculation of *Ceratonia siliqua* L. seedlings more than doubled seedlings’ survival and significantly improved seedlings’ height and collar diameter.

### AMF Drive Succession and Influence Plant Community Structure

According to Janos (1980), the mycorrhizal fungus status and the fertility of soil influence the occurrence of plant species. It is also hypothesized that AMF are drivers and as well, passengers of plant community succession (Zobel and pik, 2014). Meanwhile, the AMF status of a site determines the composition of a seral plant community, and the composition of that particular seral plant community determines the composition of infective AMF communities which will further influence the composition of the next seral plant community (Janos, 1980; Renker et al., 2004). Thus, if specific compatible relationships between certain AMF and plant taxa are required for mutual symbiont survival, the loss of compatible AMF species or individuals may limit the distribution of a particular plant species (Renker et al., 2004). Plant-soil feedback (plant-AMF feedback) is also an important concept explaining the role AMF have in succession (Kikvidze et al., 2010). Positive feedbacks promote the development of early successional communities and negative feedbacks promote plant species replacement to drive succession (Kikvidze et al., 2010).

Arbuscular mycorrhizal fungi could also potentially influence plant community structure by affecting richness or evenness of coexisting plants (Brundrett and Abbott, 2002). Only some 240 AMF morphospecies have been described forming associations with 80% of terrestrial plants (Lee et al., 2013). This indicates that AMF have no host specificness. Meanwhile, a single mycorrhizal fungus can link different plants together, thus forming mycorrhizal networks (Simard and Austin, 2010; Song et al., 2014). These networks have been shown to facilitate regeneration of new seedlings, alter species interactions, and change the dynamics of plant communities therefore, increasing plant diversity (Simard and Austin, 2010). Sowing seeds of plant species in microcosms that resembled the grassland community of the temperate zone, on soils of AMF inoculated and non-inoculated, Van der Heijden et al. (1998), Vogelsang et al. (2006), and Schnitzer et al. (2011) were able to observe that AMF inoculation improved plant community diversity by mainly increasing plants’ fitness and evenness. AMF may also be important organisms to inhibit invasion by alien species. This could be indirectly by reducing the vacant niche through increased native plants survival, establishment and diversity or can be by direct inhibition of invasives. Janos et al. (2013) reported that the presence of established extraradical mycelium prevented the survival and establishment of seedlings migrating from another ecosystem.

### AMF Increase Soil Microbial Diversity and Abundance

Arbuscular mycorrhizal fungi hyphae and root litter are the most abundant carbon source in the soil (Brundrett and Abbott, 2002). Therefore, AMF provide increased supply of energy for soil microbes to flourish. The fact that AMF influence plant communities is also considered to be one of the potential mechanisms by which AMF influence soil microbial communities (Rilling, 2004). Furthermore, AMF hyphal exudates may also stimulate microorganisms present in the mycorrhizal hyphosphere. However, the effect is variable: AMF hyphal exudates may stimulate some microorganisms but still inhibit others (Herman et al., 2012). Hence, AMF may increase the diversity and abundance of microorganisms that are beneficial to plants’ growth and health.

### AMF Effects on Plant Community Primary Productivity and Plant Competition

**FIGURE 2:**
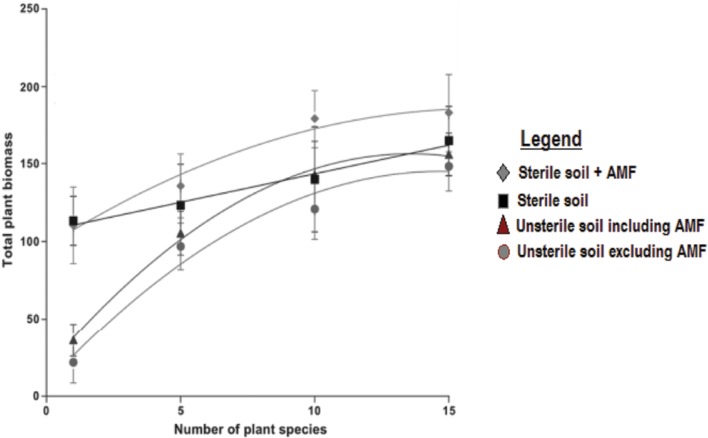
**The role of microbes and AMF in plant diversity-productivity relationship (adopted from Schnitzer et al., 2011)**.

Arbuscular mycorrhizal fungi inoculation increases plant productivity at community level and the effect increases with increase in plant species richness following the common ascending but asymptotic diversity-productivity pattern (Schnitzer et al., 2011). At low plant diversity soil microbes suppress plant productivity since their pathogenic effect increases and as the plant diversity increases, plant productivity can increase up to fivefold (**Figure 2**; circle and triangle). In the absence of soil microbes plant productivity has a weak positive linear relationship with plant diversity (**Figure 2**; square).

Sowing seeds of plant species that resembled the grassland community of the temperate zone on AMF inoculated and non-inoculated soils, Van der Heijden et al. (1998) and Vogelsang et al. (2006) were able to determine AMF effect on plant productivity at community level. Accordingly, AMF improved plant community productivity (Van der Heijden et al., 1998; Vogelsang et al., 2006). Furthermore, Van der Heijden et al. (1998) observed that increasing AMF richness resulted in increased productivity while Vogelsang et al. (2006) observed although AMF species richness increased productivity, the effect was not significant compared to single AMF inoculums effect. So, the former observation is Van der Heijden et al. (1999) argued, due to niche complementarity while the latter is Vogelsang et al. (2006) argued, due to sampling effect. The “niche complementarity” theory argues that the presence of many species and functional types results in more complete utilization of resources because different species specialize on different resources, resulting in higher overall productivity while the “sampling effect” theory argues species identity is more important than diversity and asserts that productivity increases with diversity solely due to an increased probability that communities with more species contain a few very productive species that disproportionately contribute to community-wide productivity (Schnitzer et al., 2011).

Despite the fact that the above research observations reveal that AMF increase plant productivity at community level, the recent meta-analysis based on 304 study results, which also cited the above research observation, found out that, at community level, AMF inoculation either has no effect on plant productivity or even has a negative effect (Lin et al., 2015). AMF inoculation increases plant productivity at community level only when experiments were conducted in the green house (Lin et al., 2015). Klironomos et al. (2000) were, in a greenhouse setting, able to determine the AMF effect on plant diversity and that of productivity. Accordingly, it was observed that AMF inoculation increased plant productivity but not for all AMF species. Inoculating *Glomus intraradices* N. C. Schenck and G. S. Sm. even lowered productivity compared to the non-inoculated plant community (Klironomos et al., 2000).

Similarly, variable AMF effect is observed in plant competition. Plant species competitive ability response to AMF inoculation depends on plants’ functional group, mycorrhizal status, plants’ life history (Scheublin et al., 2007; Lin et al., 2015), and also maybe below ground functional traits of the plant species (Birhane et al., 2014). The meta-analysis conducted by Lin et al. (2015) also concludes, AMF inoculation significantly increases N-fixing forbs, decreases C3 grasses and non-N-fixing forbs and woody plants, and has no effect on C4 grasses competitive ability whether these functional groups compete intra or inter-specifically (Lin et al., 2015). According to Birhane et al. (2014), in a pot experiment, AMF inoculation did not have positive effect on the competitive ability of both *Acacia etbaica* Schweinf. and *Boswellia papyrifera* Hochst. seedlings grown together. In other instances, mycorrhizal networks may result in asymmetric competition by favoring strong carbon-donor roots (Weremijewicz and Janos, 2013) or vice-versa (Walder et al., 2012).

## AMF Biotechnology for the Restoration of Degraded Lands

Degraded lands have low level of infective AMF and nursery seedlings around degraded sites may less likely be infected with sufficient AMF (e.g., Michelsen, 1992). Therefore, these sites can support the growth of late successional tree species when appropriate AMF inocula are reintroduced. Late successional tree species are obligately mycotrophic and may necessarily require AMF for their survival and fitness (Janos, 1980). More importantly, at the early stages of seedlings growth, mycorrhizal early/mid successional tree/shrub species can be even more AMF dependent than the late successional ones (Kiers et al., 2000). Therefore, AMF inoculation could potentially be considered as an important biotechnological tool in degraded lands restoration.

Arbuscular mycorrhizal fungi show no host specificity to forge symbiotic relationship with plants and are very ubiquitous, found almost in every soil (Abbott and Robson, 1991; Brundrett and Abbott, 2002; Barea et al., 2011; Al-Karaki, 2013). Hence, many researchers argue that AMF inoculation is likely to be valuable in only few conditions such as mine fields where indigenous AMF inoculum is surely little or none available (Brundrett and Abbott, 2002). Koide and Mosse (2004) suggested instead of going for AMF inoculation it would be quite economical and appropriate to focus on managing the indigenous AMF population of a site. According to Renker et al. (2004), inoculation is an important but the last option. However, contrary to having several dispersal agents such as; wind, water, rodents, birds, worms, and ants (Brundrett and Abbott, 2002), AMF were observed to have poor dispersal. Accordingly, Hailemariam et al. (2013) were able to observe that within a single piece of farm land, soil AMF status and infectiveness can vary in short distances indicating poor dispersal. Similarly, Friese and Allen (1991), also indicated that AMF have poor dispersal. Therefore, to overcome the dispersal limitation of AMF, inoculation may be a worthily intervention.

Meanwhile, AMF inoculation has proved to be effective under wide range of soil conditions (Janos, 1980; Brundrett and Abbott, 2002) including on soils with good AMF abundance (e.g., Banerjee et al., 2013). Positive AMF effect is not ensured by the presence of abundant indigenous AMF but by both abundance (quantity) and efficiency (quality) of indigenous fungal populations (Onguene and Kuyper, 2005). Veiga et al. (2011) also demonstrated that AMF inoculation suppressed weeds and, interestingly enough, hypothesized that AMF inoculation could suppress ruderal plants which are known to invade degraded sites (Veiga et al., 2011). This is particularly important in ecological restoration since ruderal plants could invade degraded lands and compete with tree/shrub seedlings planted.

If the importance of AMF inoculation in the restoration of degraded lands is agreed, the next question to ask will be; what kind of inocula should be prepared? AMF show wide range of functional diversity (Johnson et al., 1997; Klironomos, 2003; Smith et al., 2011) and their effect is within the mutualism-parasitism continuum (Johnson et al., 1997). Likewise, Hoeksema et al. (2010) summarized that certain plants functional groups viz. non-N-fixing forbs and woody plants and C4 grasses show more positive responses to AMF inoculation. Klironomos (2003) also demonstrated that exotic-native AMF strain-host or vice-versa combination results in highly parasitic interaction. Therefore, deciding on the type of inoculum to prepare is a very important step. Based on the currently available data, the use of native inocula should be preferred to the use of exotic inocula. Early seral AMF should be used when seedlings are inoculated for restoration, even for late seral tree species (Allen et al., 2003). Late seral AMF have big spores and demand much carbon and hence, seedlings may not benefit from them. Instead, seedlings benefit from early successional AMF which are usually having small spores and smaller carbon demand (Allen et al., 2003). Likewise, the use of inocula from grasslands is promoted. AMF abundance in grasslands can be more than tenfold than that of in the forestlands and AMF from grasslands do have significantly high inoculation effect (Fischer et al., 1994). That was why Onguene and Kuyper (2005) applied fresh grassland whole-soil inoculum on various soils and three tree species seedlings. According to the result Onguene and Kuyper (2005) obtained, although early successional grassland inoculum had positive effect for most of the cases (80%), the fact that it is an inoculum from grassland resulted in significantly negative effect on *Terminalia superba* Engl. and Diels seedlings grown on agricultural and early successional forest soils. Hence, Onguene and Kuyper (2005) concluded; allochthonous AM inocula may not be always effective. Hence, the use of planting site adapted AM inocula may be recommended. The other reason for the observed negative effect may also be related to host plant’s fungi preference (Onguene and Kuyper, 2005). There are data to demonstrate that inocula from conspecific source show better affinity to the host plants’ root (e.g., Kiers et al., 2000). Similarly, there are data to show that plant species even that do co-occur may prefer to associate with distinct AM fungi communities (e.g., Wubet et al., 2006; Davison et al., 2011). There are also data to show that distinctively different AMF communities colonize seedlings’ and adults’ roots of a single tree species (e.g., Wubet et al., 2009). Therefore, one has to ask; does inoculating seedlings with AM inocula from seedlings’ rhizosphere or adults’ deliver better positive effect? Kiers et al. (2000) have found out that although conspecific inocula from adults had better affinity to inoculated seedlings, the effect on their growth was mostly relatively small showing that, inocula even from conspecific adults, may not be suited for seedlings inoculation.

Selecting few of the dominant planting site adapted AMF species, multiplying them and applying as inocula may not be also a very good idea specially when there are established AMF in the planting site. Increasing the density of few of the dominant AMF species and applying as inocula had resulted in negative effects on plant growth by disrupting indigenous AMF community structure and thereby creating competition among AMF to ultimately result in inoculum failure (Janoušková et al., 2013). Therefore, in areas with low levels of indigenous AMF abundance, multiplying all not only the dominant AMF species and applying all may be the best option.

The AMF richness in AM inocula is considered to improve inocula effectiveness. Plant response is substantially lower when inoculated with single AMF species and the response keeps increasing from multiple fungal species to whole-soil inoculums (Hoeksema et al., 2010). Likewise, Barea et al. (2011) compiling long years of experience in AMF research recommend the use of autochthonous foundation shrub inoculated with autochthonous AMF consortia inoculums to best restore degraded lands of the Mediterranean. The shrub not only acts as a foundation species but also serves as a resource island for AMF (Barea et al., 2011). However, not all ecologists agree by the application of AMF species rich inocula; some argue that better results due to inocula with better AMF species richness is due to sampling effect and selecting single effective AMF species should get the attention of restoration ecologists. Sampling effect is discussed earlier.

The other challenge associated with AMF biotechnology is related with inocula production for large-scale application. This is due mainly to the obligate nature of AMF. Meanwhile, AMF cannot be cultured axenically (Azcón-Aguilar et al., 1999; Fortin et al., 2005) and host plant based AMF multiplication is mandatory. These host plant based conventional inocula production methods (substrate based pot culturing and substrate free methods of hydroponics and aeroponics techniques) are costly and large scale production of AMF inocula may hardly be possible. Effective monexenic *in vitro* culturing of AMF has been made possible few decades ago (Bécard and Fortin, 1988) and in India, using this method, large-scale industrial production of biologically clean AMF inocula was possible (Adholeya et al., 2005). Readers are directed to read Adholeya et al. (2005) and Cranenbrouck et al. (2005) to grasp the potential and the technique of monoxenic *in vitro* AMF culture production for large-scale application. Readers are also directed to read Azcón-Aguilar et al. (1999) to get proper definitions of axenic and monoxenic cultures.

However, until now, monexenic *in vitro* culturing is not widely practiced. This is due mainly to the fact that; (1) undesired contamination is hardly avoidable and the technique is technology and skill demanding (Bago and Cano, 2005), (2) there are ethical and legal concerns, and (3) it is rather very hard to identify each genotype (even morphotype) hence, most if not all, AMF are not readily culturable (Fortin et al., 2005). AMF momoxenic *in vitro* culturing uses transformed [using *Rhizobium rhizogenes* (Riker et al.) Young et al.] hairy roots as host owing to the fact that these hairy roots are better suited than the non-transformed hairy roots since they grow on hormone free media and without developing shoots and leaves (Puri and Adholeya, 2013). Meanwhile, AMF monoxenic culture as it is practiced now could potentially be challenged with biosafety related issues.

Due to the lack of cheap and easy AMF inocula production for large scale application, managing the *in situ* AMF is sometimes considered to be an effective AMF biotechnology for the restoration of degraded lands. The meta-analysis by Lekberg and Koide (2005) showed that short fallow could be as good as inoculation to improve plants growth and productivity. It was shown that an obligately arbuscular mycorrhizal pioneer nurse shrub *Lavandula stoechas* L. improved the field survival and establishment of *Cupressus atlantica* Gaussen seedlings by increasing, among others, *in situ* infective AMF abundance (Duponnois et al., 2011). Kumar et al. (2010) also compared different plant composition effects on *in situ* management of AMF on a degraded coal mine spoil. Accordingly, they demonstrated that using cover crops mainly grasses and N-fixing shrubs in the plant composition, significantly enhanced AMF abundance, diversity and infectiveness. Hence, AMF can be manipulated by fallowing or/and by designing the plant species composition to ultimately result in increased AMF abundance which intern facilitates restoration. However, some investigations indicated that grass cover can significantly suppress individual tree/shrub seedlings-saplings growth (Riginos, 2009) or may have variable seasonal effects (Good et al., 2014). Therefore, investigation on cover plant management options to effectively manage AMF and facilitate tree/shrub seedlings growth can be an important research topic.

**FIGURE 3:**
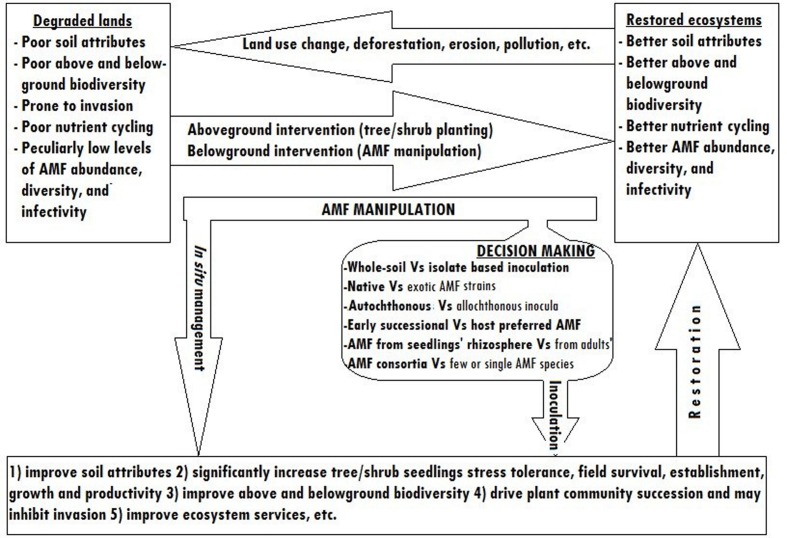
**Simplified schematic model showing degraded lands restoration through AMF manipulation**.

Nowadays, substrate free inocula preparation methods and *in vitro* production on excised plant roots are being intensively researched to make AMF inoculation less costly (Ijdo et al., 2011). The pot culture inocula preparation method, although it is labor intensive and costly, can be a source of employment especially in developing countries. Therefore, pot culture based AMF biotechnology will remain to be a feasible way of degraded lands restoration in most parts of the world. **Figure 3** shows the simplified schematic model of degraded lands restoration using AMF.

## Conclusion

This review paper has compiled facts regarding the AMF role in the above and belowground ecosystem processes relevant to ecological restoration. Accordingly, it is possible to conclude that AMF; have a well documented positive role in nutrient cycling and improved soil attributes. AMF also improve plants’ tolerance to biotic and abiotic stresses, and significantly increase tree/shrub seedlings survival, establishment and growth. AMF play pivotal role in plant community succession and may directly or indirectly prevent invasion by alien plant species. At plant community level, AMF increase both above and below ground biodiversity but their effect on primary productivity maybe low. The AMF effect on plant competition is also variable and mostly negative. Available data as of yet, indicate that there are very few outfield experiments done on AMF effects on tree/shrubs seedlings survival and establishment. This review was not also able to clearly trace a research result showing the AMF effect on the competitive ability of tree seedlings planted with annual and perennial grass and/or herbaceous weeds. Based on the currently available data, however, it can be concluded that AMF inoculation can significantly increase the success of degraded lands restoration and for better results reducing competitors and seedlings density (increased seedling spacing) is recommended.

Based on the data reviewed in this article, we recommend for future AMF effect researches to give emphasis to outfield experiments. The AMF effect on the competitive ability of tree seedlings compared with annual and perennial herbaceous weeds should be investigated. Data reviewed here showed that almost all research observations conducted on AMF effect at community level are on microcosms of grasslands; and mainly temperate grasslands. Future researches should focus on forest communities of both the temperate and tropics. For an effective large scale application of AMF inocula biotechnology, pot based inocula multiplication will remain to be significantly cost ineffective. Therefore, investigating and researching on cost effective multiplication methods of substrate free and *in vitro* culture and/or optimization of the effects of low-cost fresh AMF inoculation techniques like using grassland top soil or managing AMF *in situ* using several cover crops including grasses need further attention in the future. Optimization of monoxenic *in vitro* AMF culture products and using non-transformed hairy root organ could also be an important research area until axenic *in vitro* AMF culturing is ultimately made possible.

## Author Contributions

FA did all the data gathering and write-up. TB and EB considerably contributed intellectually by providing comments and guidance at every milestone of the manuscript development. All authors approved publication of the article.

## Conflict of Interest Statement

The authors declare that the research was conducted in the absence of any commercial or financial relationships that could be construed as a potential conflict of interest.

## Footnotes

1 https://www.cbd.int/sp/targets/

2 http://www.wri.org/faq/what-degraded-land
